# Male Reproductive Cancers and Infertility: A Mutual Relationship

**DOI:** 10.3390/ijms16047230

**Published:** 2015-03-31

**Authors:** Eva Tvrda, Ashok Agarwal, Nawaf Alkuhaimi

**Affiliations:** 1 Center for Reproductive Medicine, Cleveland Clinic, Mail Code X-11, 10681 Carnegie Avenue, Cleveland, OH 44195, USA; E-Mails: evina.tvrda@gmail.com (E.T.); nawafalkuhaimi@gmail.com (N.A.); 2 Department of Animal Physiology, Faculty of Biotechnology and Food Sciences, Slovak University of Agriculture, Tr. A. Hlinku 2, 94976 Nitra, Slovakia; 3 College of Medicine, Alfaisal University, P.O. Box 50927, 11533 Riyadh, Saudi Arabia

**Keywords:** male infertility, testicular cancer, prostate cancer, genetics, epigenetics, environment, fetal environment, relationships

## Abstract

Reproductive dysfunction and malignancies related to the male gender represent a serious health concern, whose incidence has significantly risen over the past years. Prior to treatment, testicular or prostate cancer patients often display poor semen characteristics similar to subfertile or infertile patients. This fact is underscored by cases where the malignancy is often diagnosed in males who undergo a general fertility screening. This review aims to examine the associations between male infertility and reproductive cancers focusing on common etiologies and biological mechanisms underlining these pathologies. Furthermore, we discuss compelling epidemiological data hypothesizing that male reproductive failure may act as a precursor of future andrological malignancies, including testicular or prostate cancer, thus providing a stimulus for a more specific research in male reproductive health and emphasizing the importance of this relation for physicians taking care of male patients with a reproductive disease.

## 1. Introduction

The diagnosis of infertility is implied when a couple has continuous unprotected intercourse for at least one year without being able to conceive. Approximately 15% of these couples experience infertility, although this prevalence intensifies with increasing age. In 20% of the cases male factor is solely responsible for reproductive complications, while it will partially contribute to an additional 30% of infertile couples [1,2,3]. Male reproductive dysfunction has received substantial interest from academics and researchers over the past few decades. It is now widely accepted that reproduction in men may be compromised by underlying tumors exclusive to the male gender, including testicular and prostate cancer. Nevertheless, current research has attempted to understand if male reproductive failure may precede reproductive malignancies, and to provide a comprehensive view on the mutual associations connecting reproductive dysfunction and cancer bound to the male gender. The aim of this review is to investigate and interrelate common causes, risk factors, and molecular mechanisms linking compromised male fertility status with testicular or prostate cancer. Furthermore, we will focus on examining the hypothesis of male infertility acting as a predecessor of male reproductive cancers.

## 2. Reproductive Cancers

### 2.1. Testicular Cancer

Even though testicular cancer (TC) is not ranked as a prevalent male cancer in the world, it is the most common oncological diagnosis in the reproductive age group of patients (20–35 years). The theory behind this critical connection possibly lies in a burst of hormones typical for males reaching puberty, triggering carcinoma *in situ* (CIS) cells, being “innate” and having the potential to proliferate into cancer cells [4]. Different types of TC exist, however the most common is testicular germ cell cancer (TGCC), representing about 95%, with approximately 9000 diagnoses in the United States each year. TGCCs are of two types, seminomas and non-seminomas. Each constitutes approximately 50% of TGCCs, while 15% of patients present with both types [5].

Seminomas originate in the germinal epithelium of the seminiferous tubules, where malignant cells most likely arise from primordial germ cells (PGCs)—progenitors of the gametes [6]. Compared to other TGCCs, these tumors are generally more responsive to treatment via orchiectomy, chemotherapy or radiation. Unlike seminomas, nonseminomas represent other types of germ cell tumors, such as embryonal carcinoma, teratoma, yolk sac tumor, choriocarcinoma, and are usually treated with chemotherapy due to a lower sensitivity to radiation.

**Figure 1:**
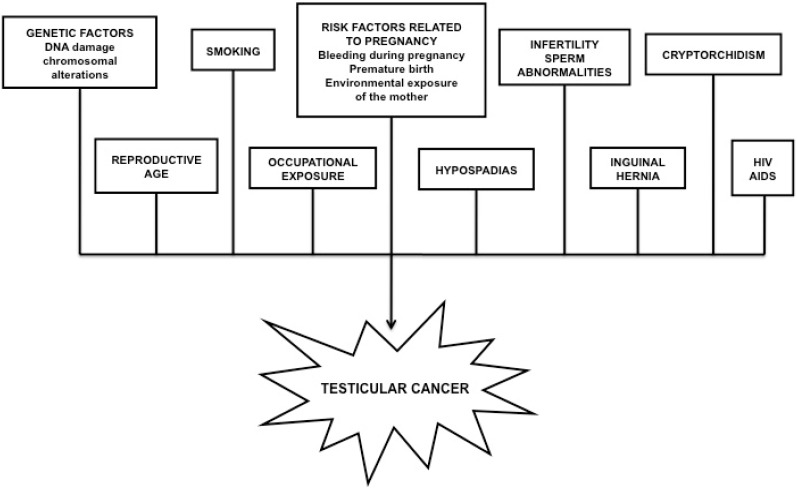
The most common risk factors contributing to testicular cancer development.

A small percent of testicular malignancies include stromal tumors such as Leydig cell and Sertoli cell tumors, as well as other rare or poorly characterized histologic types [4]. The most efficient and cost effective method of prevention is auto-palpation of the testicles [7]. Treatment of TC depends on the type and severity of the disease, medical care varies from an appropriate chemotherapy and radiotherapy regime to orchiectomy. The most common risk factors contributing to TC development are summarized in Figure 1.

### 2.2. Prostate Cancer

According to the Centers for Disease Control and Prevention, prostate cancer (PC) is the most common cancer found in American men (128.3 out of 100,000), and it is the second leading cause responsible for mortality in the United States [8]. There are various types of prostate tumors, and the malignancy often develops in diverse parts of the organ. The precursor to prostate cancer—the prostatic intraepithelial neoplasia—usually originates in the peripheral zone of the organ.

Acinar adenocarcinoma is the most frequent form of PC and accounts for 90%–95% of the cases. This tumor develops from the cells lining the prostatic glandular tissue, responsible for the secretion of the prostate fluid. Other types of prostatic adenocarcinoma include atrophic, foamy, colloid or signet ring carcinoma [9]. The remaining 10% of PC cases represent rare tumor types, such as transitional cell (or urothelial) cancer, squamous cell cancer, small cell cancer or sarcoma [10].

**Figure 2:**
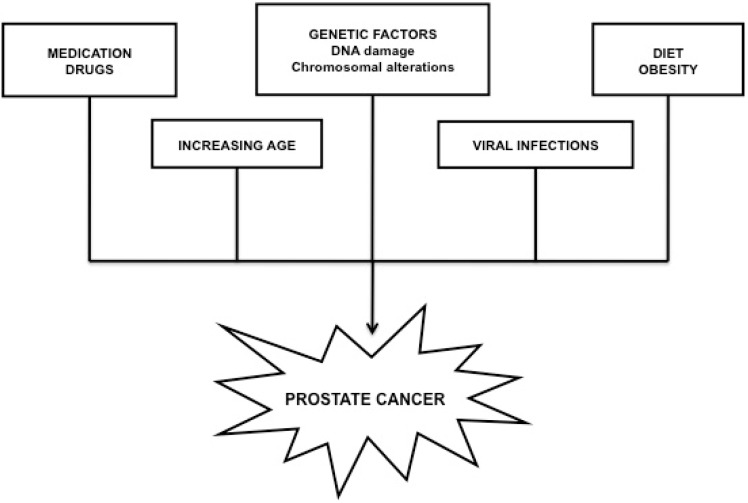
The most common causes contributing to prostate cancer development.

PC is considered to be highly fatal because if left untreated the malignancy will spread fast through the venous plexus of the prostate and eventually find its way to the vertebra followed by retroperitoneal and brain metastases. More than 25% of the cases present with metastatic disease at the time of diagnosis [11]. There is not a universal strategy for PC prevention, although regular check-ups, appropriate life style and feeding habits, as well as a minimal exposure to hazardous materials may significantly reduce the risks of future carcinogenesis. Treatment options are generally adjusted to the stage and characteristics of the tumor, and may include radiation and hormonal therapy, chemotherapy, surgery or cryotherapy [12]. Generally accepted causes for PC development are shown in Figure 2.

## 3. Causes and Factors Governing the Association between Male Infertility and Reproductive Cancers

### 3.1. Genetic Factors

Genetic factors play a critical role in the development of both male cancers as well as a compromised fertility.

Surprisingly, genetic alterations alone contribute to about 25% of the causes for TC [13]. The remaining 75% can be attributed to yet unknown factors. Around 200 genes are to be found on the Y chromosome, with most of the sex determining genes located on the short arm of the chromosome. Changes and mutations in the SRY gene are considered to cause both gonadal dysgenesis as well as tumor development [14]. It is believed that the cells of TGCCs are derived from primordial germ cells [15,16] with alterations to the DNA sequence in the Y chromosome [17]. Cases of both reproductive dysfunction as well as TC occurrence have also been associated with alterations in DNA repair genes and tumor suppressor genes. Chronic oxidative stress often reported in infertile patients is indicative of a deficiency in DNA repair pathways, leading to unstable genomic components related to diverse cancer types [18,19]. A typical tumor suppressor gene susceptible to damage is the p53 gene, essential for tumor prevention, genomic stability and stress response. p53 is furthermore crucial for a proper course of meiosis within the primary spermatocytes. Mutations of p53 may lead to chromosomal and genomic instability, increasing the probability of cancer cell development and additional mutations [20]. At the same time, this instability often leads to a compromised reproduction potential as seen in animal studies, according to which p53 knockout mice are infertile [21].

TGGCs more commonly arise due to alterations in chromosomal structure rather than changes in the actual DNA sequence. Examples for chromosomal alterations include changes to the 12p region, which are usually associated with TC development as well as impaired spermatogenesis [22,23,24]. Amplification of the genetic region 12p has been shown to increase the risk for seminoma germ cell cancer (SGCC) development, while additions in the 17q region or deletions in the 10q region are associated with an elevated incidence of non-seminoma germ cell cancers (NSGCC) [15].

Abnormalities of the Y chromosome have been suggested to serve as a link between male infertility and PC [25,26]. While Y microdeletions are believed to be the major genetic cause for oligozoospermia or azoospermia [27,28], a study conducted at the University of California [29] showed heterogeneous and differential expression patterns of the Y chromosome genes from prostate tumor samples, suggesting that over- or underexpression of several Y chromosome genes may play a role in an abnormal endocrine stimulation of PC cells. A different genetic abnormality often reported in patients suffering from infertility or PC, are alterations in the CAG repeats in genes encoding the androgen receptor (AR). A patient study conducted by Mosaad *et al.* [30] revealed significant changes in the CAG repeat length among fertile and infertile subjects, proposing the role of this genetic alteration in a reduced AR activity, translated into oligozoospermia or asthenozoospermia [31,32]. At the same time, variations in the CAG repeats have been associated with aggressive types of PC [33,34,35]. On the other hand, mutations to the kallikrein-related (KLK) protease gene family may result in abnormal secretion of diverse serine proteases, including the prostate-specific antigen (PSA), a well-known marker for male infertility and PC screening [36]. Mutations in repair genes have also been associated with the occurrence of both male reproductive dysfunction and PC. Meiotic arrest, resulting from deficient DNA repair mechanisms, may lead to azoospermia [37,38,39], while polymorphisms of the MSH3 gene participating in the mismatch repair (MMR) system have been interrelated with PC manifestation [40]. According to Walsh [3], transcriptional errors in repair genes reported in both germ-line as well as somatic cell DNA, could originate from one source, thus providing a suitable genetic link between PC and male infertility.

Genetic disorders may represent an additional foundation for male infertility and reproductive cancers. Patients diagnosed with Mixed Gonadal Dysgenesis (45X/46XY) are at increased risk for gonadal tumors, impaired fertility, and fibrosis as a result of ongoing structural changes within the reproductive tissue [41]. Genetic conditions related to poor testicular development often observed in patients diagnosed with the Down (47, XX/XY, +21) [42] or Prader-Willi syndrome (15q11-13 deletions) [43] may lead to elevated risks for TC occurrence. On the contrary, it was postulated that men suffering from the Klinefelter syndrome (47, XXY)—the most common sex chromosome disorder in males affecting approximately one out of every 600 men [44] have lower chances for developing a prostate malignancy [45] due to hypogonadism and chronically low circulating androgen levels [46]. This hypothesis was proven correct by an epidemiological study showing a relatively lower PC incidence among patients diagnosed with this sex chromosome anomaly [47], followed by two case studies reporting on PC development in Klinefelter patients treated with testosterone or androgen replacement therapy [48,49].

### 3.2. Epigenetic Factors

Unlike genetic factors, epigenetic mechanisms do not directly alter the DNA sequence or quantity [50]. Instead, epigenetic mechanisms affect the regulation by enhancing or silencing of gene transcription via DNA methylation, histone modifications or histone-protamine interactions.

DNA methylation, which is the most widely studied mechanism of epigenetics, is a collective term that encompasses methylation or demethylation of the methyl group located on the 5' end of cytosine in the DNA sequence. Histone modification includes different changes such as acetylation, methylation, ubiquitination, and phosphorylation of histones in compacted nucleosomes. Histones are believed to be the best transporter for epigenetic information as these proteins have a major impact on the chromatin structure and transcriptional activity [51,52]. Both DNA methylation and histone modification are important epigenetic mechanisms of gene regulation playing critical roles in male reproductive dysfunction individually as well as cooperatively. Aberrant epigenetic events including DNA hypo- and hypermethylation together with altered histone acetylation have been associated with a decreased spermatozoa concentration, motility and morphology in a multitude of studies [53,54,55,56,57,58]. Similar epigenetic mechanisms have been proposed to be involved in the development of multiple malignancies, including testicular and prostate cancer [59,60,61].

Changes in the expression patterns of DNA methyltransferases (Dnmts) and histone methyltransferases (HMTs) at the spermatocyte level [62] are believed to be the leading epimutations related to transcriptomic profiles in testicular as well as germ cell tumors [63]. Compared to only a few oncogenes known to be activated by DNA hypomethylation, a large number of tumor suppressor genes are transcriptionally silenced by DNA hypermethylation in cancer cells. Seminoma PGCs represent a typical cell type where DNA methylation and parental imprints are erased, restoring totipotency [64]. At the same time, genes of pluripotency are particular targets for epigenetic modifications in testicular malignancies, as both early fetal germ cells as well as undifferentiated germ cell tumors express pluripotency markers such as the Nanog and Oct3/4 transcription factors [65]. While the Nanog promoter has been shown to be hypomethylated in spermatogonia and hypermethylated in spermatozoa [66], its methylation in germ cell tumors strongly correlates with the differentiation state of the malignant cell. At the same time, it has been reported that the CpG islands present in the Oct3/4 transcription factor showed signs of hypomethylation in seminomas and embryonal carcinomas [67].

Recent data indicate a significant epigenetic link between male infertility and PC [68]. Aberrant DNA methylation is the most common epigenetic alteration in PC, leading to genomic instability and incorrect gene expression [3]. An extensive review by Park [69] identifies more than 30 genes undergoing aberrant epigenetic methylation related to prostate cancer development. These genes include classic and putative tumor-suppressor genes as well as genes involved in diverse molecular pathways such as hormonal responses, tumor-cell invasion, cell cycle control or DNA damage repair, a variety of which play important roles in the process of spermatogenesis [3]. Global and locus-specific changes in chromatin remodeling, altered activity of histone-modifying enzymes as well as microRNA deregulation are additional epigenetic changes proposed to be associated with prostate dysfunction and carcinogenesis, disruption of AR signaling pathways and cell death [3].

Finally, it has been shown that assisted reproductive technologies (ARTs) could cause epigenetic changes [50], arising from the use of spermatozoa with incomplete reprogramming, or *in vitro* procedures while epigenetic changes are still taking place [70], compromising a proper embryogenesis, fetal development and health of future offspring.

### 3.3. Environmental Factors

Humans are exposed to diverse environmental agents potentially hazardous to the reproductive system. Male reproduction is known to be highly responsive and sensitive to a variety of chemical and physical agents generated by industrial and agricultural activities [71]. Such risk factors are frequently found in the general environment as well as in occupational activities.

Numerous case-control and ecological reports emphasize on a strong correlation between acute or chronic exposure to heavy metals, pesticides, organochlorides as well as radiation and a higher incidence of TC (reviewed by Béranger *et al.*) [72] or PC (reviewed by Doolan *et al.*) [73] along with congenital anomalies, such as cryptorchidism [74] or hypospadias [75] and a notable decline of male fertility in the general population [76,77,78].

Male reproductive dysfunction as well as reproductive cancer occurrence in the general population has attracted increasing attention due to studies linking male reproductive diseases to the widespread use of chemicals with hormonal properties—endocrine disrupting agents (EDAs) [79]. EDAs are known to act as imperfect ligands—agonists or antagonists, to both nuclear and membrane receptors for hormones, therefore interfering with hormone-regulated cell signaling and gene expression [80]. EDAs act through different mechanisms of action, with synergistic or antagonistic outcomes [81]. Many endocrine disrupting chemicals have estrogenic or anti-androgenic activity, thus interfering with the estrogen receptors (ER) or the androgen receptor (AR). Orphan receptors may be another target for EDAs, notably the aryl hydrocarbon receptor (AhR)—a transcription factor for detoxifying enzymes [82]. Disruption of the AhR activity leads to the degradation of sex steroid receptors [83]. Finally, diverse EDAs are able to alter the hormone bioavailability by interfering with its secretion and transport or to disrupt the enzymatic pathways involved in hormone synthesis or metabolism [84,85].

The group encompassing EDAs is complex and embraces ubiquitous synthetic substances used in the industrial and agricultural sector including lubricants and solvents, plastics, plasticizers and drugs [86]. Natural products such as genistein [87] and certain heavy metals [88] can also exhibit endocrine-disruptive effects.

Diverse epidemiological studies (reviewed by Aitken *et al*.) [89] emphasize on a worldwide decrease of semen quality and a higher incidence of male reproductive malignancies suggesting that both conditions may share an endocrine etiology.

The Testicular Dysgenesis Syndrome (TDS) is the most accepted epidemiological correspondence between low semen quality and an increased prevalence of cryptorchidism, hypospadias and TC, probably arising from perinatal disruption of a proper testicular development and function [90] as a consequence of exposure to EDAs [79]. Skakkebæk *et al.* [90] propose that TDS originates from conception and may result in a cascade of defects primarily affecting Sertoli and Leydig cells.

Although specific mechanisms of action are poorly understood, it is known that EDAs cause an increase in estrogen levels, inhibiting the hypothalamo-pitutary-gonadal axis, leading to a decreased production of follicle stimulating hormone (FSH) and subsequently, altering the replication process of the Sertoli cells [21]. TDS is thus associated with a decreased Sertoli cell number, lower levels of anti-Mullerian hormone, and a decreased expression of SRY, resulting in abnormal sexual differentiation, hypospermatogenesis [91,92], and hormonal carcinogenesis [93,94]. Furthermore, Sertoli cells are believed to be the primary cellular structure for EDA accumulation, magnifying the effects of endocrine disruption over an extended period of time [86].

Impaired Leydig cell function is another cellular characteristic of TDS [95,96]. In mild cases, patients often present with low testosterone levels, decreased testicular volume, and poor semen quality, while severe cases face an increased risk for hypospadias, cryptorchidism and TC [97].

The susceptibility of an expecting mother to endocrine disruptors may come hand in hand with epigenetic changes to primordial germ cells (PGC), inhibition of gene expression and suppression of cell proliferation. Later in life, even when EDAs are no longer present, PGCs may proliferate and eventually become the baseline cells of TGCCs [98].

While precise interrelations between endocrine disruption and the male reproductive function are still insufficiently understood, EDAs have been strongly correlated with each of the reproductive health problems associated with TDS in both human and animal studies. Epidemiological data hypothesize that *in utero* exposure to environmental levels of EDAs is connected to an increased risk for a later presentation of typical TDS characteristics, including a substantial decrease in semen quality [99,100,101]. At the same time, EDA exposure has been proposed to be a prominent factor providing an explanation to a number of idiopathic infertility cases [78,102,103]. Moreover, permanent contact with certain types of EDAs, including pesticides and chlorinated biphenyls has been shown to increase both seminoma and nonseminoma germ cell cancers [104]. Geographical location, and thus a potential exposure to different industrial or agricultural chemicals is also a well-known risk factor for TC [105]. Lastly, the importance of treating TDS symptoms before adolescence is showcased in a study conducted by Walsh *et al.* [106] revealing that males who had their cryptorchidism treated after the age of 13 had twice the risk of developing TC compared to males who had the treatment before the age of 13.

Increasing number of epidemiological and experimental studies provide evidence that specific EDAs may have a significant impact on the development or progression of prostate cancer. According to Alavanja *et al.* [107] and Mahajan *et al.* [108] a variety of pesticides display inhibiting activities on the P450 enzyme superfamily involved in the intraprostatic metabolism of steroids, drugs and nutrients [109,110]. Endocrine disruption may thus alter the steroid balance and availability contributing to increased PC incidence. Data from rodent models and human prostate cell lines indicate that early life exposure to Bisphenol A (BPA) may increase the susceptibility to hormonal carcinogenesis in the prostate gland, possibly by genetic or epigenetic reprogramming [111]. On the other hand, Polychlorinated Biphenyls (PCBs) are able to transform prostate cells by disruption of the cellular gap junctions and increased genomic imbalance through double-stranded DNA breaks [112]. Different drugs with endocrine disrupting effects lead to premature acinar atrophy and aging-associated prostatitis [113]. This observation may be particularly significant in light of recent evidence that chronic inflammation may play a critical role in PC initiation [114].

Because of the androgen sensitivity of the prostate gland, the TDS theory is applicable to prostate cancer as well. Following abnormal gonadal function, prostate—an androgen sensitive organ may not receive enough or adequate differentiating signals during critical stages of development, thus increasing the risk for a prostate malignancy [3,115].

It is important to remember that these etiologies are not completely independent, and are interrelated. The more risk factors a male has the more susceptible he is to eventually develop TC or PC. Interconnections between individual risk factors have been proven in studies with monozygotic twins, which exposed to different environments exhibited epigenetic variations in spite of identical genetic information [116]. This provides us with evidence that environmental, epigenetic, and genetic factors play a role in affecting each other. Racial backgrounds have also been shown to be related to the intensity of risks associated with TC, and Hispanics have been demonstrated to be the most prone to testicular tumor development in the United States. This observation may be dependent not only on genetic but also on environmental circumstances. The initial genetic predisposition and exposure to organochlorides have been documented to increase TC development [65]. At the same time, EDAs have been proven to cause epigenetic mutations that are persistent and transmitted to the offspring [117,118]. Both cancer and spermatogenic defects have been reported to have the ability of this transgenerational inheritance [118].

Different causes for male cancer development present with a diversity of effects. Some will cause cancer upon exposure, others will force cells into a “hibernation state” and when puberty is achieved alongside with an increased endocrine activity, these cells will eventually “awaken” and proliferate into cancer cells. Since infertile patients present with abnormal reproductive characteristics due to different etiologies, they might also be carrying the “hibernating” cells. These observations have initiated the hypothetic postulation that infertile men may be more susceptible to develop cancerous tumors.

## 4. Infertility: A Precedent of Male Reproductive Cancers?

It has been well documented that cancer as a pathological process may exhibit a variety of deleterious effects on male fertility, even before any treatment has been administered [119,120]. The most common malignancy-associated effect reported is the disruption of the hypothalamic-pituitary-gonadal axis, leading to serious alterations to a delicate endocrine balance within the male reproductive system [121,122]. Systemic effects of a tumor presence include a direct immunological or cytotoxic injury to the germinal epithelium [119,123,124], leading to significant alterations of spermatogenesis [124] and generation of antisperm antibodies [125]. Fever and malnutrition commonly observed in cancer patients may be associated with spermatogenic alterations, leading to a severely diminished sperm concentration or even azoospermia [126,127]. Psychological effects including anxiety and depression have been associated with infertility in many oncological cases. Sexual dysfunction and fertility distress are known to be long-term consequences proceeding for years after the actual cancer diagnosis [128]. All the above-indicated pathological changes may individually or collectively lead to decreased semen quality and fertility impairment, being often present at the very time of diagnosis [119,120].

Given shared etiologies, risk factors and molecular pathways, recent attention has been placed on the question if male reproductive dysfunction may precede testicular or prostate cancer. The remaining section of this review will therefore discuss the currently available research data interconnecting these diseases, limitations of the studies, as well as possible mechanisms emphasizing on their mutual relationship.

The development of TGCCs due to infertility has been studied in the past. It is believed that TGCCs could arise in men with underlying carcinoma *in situ* (CIS) cells. Pryor *et al.* [129] studied the relationship between infertility and TC development. Out of a pool of 2043 men, the study identified 8 patients presenting with CIS cells. 6 out of the 8 subjects eventually developed TGCCs later in life. Different reports suggest that the presence of CIS cells may lead to both infertility through impaired spermatogenesis as well as cancer as these cells have a genetic stem cell profile and pluripotent characteristics carrying the potential to proliferate into TGCCs [15,130], supporting the idea that TC and infertility share similar environmental, genetic, and ethnic backgrounds [131,132]. Petersen *et al.* [133] provided additional evidence that patients diagnosed with TGCC and CIS cells usually present with abnormal semen parameters. A Danish study by Jacobsen *et al.* [134] looked into a pool of patients seeking infertility treatment at a sperm analysis laboratory in Copenhagen. The investigation found that out of the 32,442 cases, 89 progressed to develop TC, meaning that men with abnormal semen parameters or infertility were at a 1.6 times higher risk to develop TC in comparison with healthy men. Out of the 89 cancer cases, 50 developed seminomas, 37 had nonseminomas and 2 were diagnosed with an unspecified testicular malignancy. Additionally, the study reports that men were more likely to be diagnosed with cancer within 2 years after the first semen analysis, suggesting that abnormal semen parameters were indicative of future risk of TC development. In the case of azoospermic patients, those who had no children faced a 3.65 higher chance of eventual TC development when compared to azoospermic patients with children. Furthermore, men with spermatozoa concentrations ranging from 0 to 20 million were more susceptible to TC occurrence compared to men with concentrations greater than 20 million (a standardized incidence ratio 2.3 *vs.* 1.1, respectively). It is however important to keep in mind that both patient groups ultimately faced the threat of possible TC development. At the same time, men with abnormal sperm motility were 2.5 times more susceptible to TC, while men with abnormal morphology had a 3 times higher risk of testicular tumor development. A combination of different semen abnormalities led to a drastic increase in a possible TC diagnosis, as two joint subfertility parameters resulted in a 2.7 times higher risk, while a fusion of all three abnormalities was associated with a 9.3 higher chance of future testicular tumor development. It is therefore important to acknowledge that patients diagnosed with 3 subfertility measures faced a 9-fold increase in eventual TC diagnosis. These results prove that suboptimal semen parameters could be a precursor as well as a predictor for TC, thus they should be taken seriously. As discussed in the study, Jacobsen *et al.* [134] assume that a hypothetical removal of patients with infertility caused by a female factor would lead to even more significant differences. Doria-Rose *et al.* [135] brought ingenuity to their methodology as instead of observing infertile men and conducting regular follow-ups, they looked directly at TC cases and subsequently used a “backtrack” strategy using the National Cancer institute’s Surveillance Epidemiology and End Result (SEER) in order to see whether or not the patients had been diagnosed with infertility prior to TC development. Out of 329 men, 183 were diagnosed with seminomas and 146 had nonseminomas—a finding consistent with the outcomes by Jacobsen *et al.* [134]. 329 TC cases were more likely to have been previously diagnosed with infertility or had cryptorchidism. The study showed that the testicular malignancy was 2.4 times more likely to have been interrelated with previous infertility. The most important data collected from this study included a reduced risk of TC development in men who had previously fathered children. The odds ratio was 0.76 however the limitation of this observation was based on a comparative analysis with infertile men and patients diagnosed with cryptorchidism. Excluding cryptorchidic cases, the risk ratio was adjusted to 0.82 and to 0.87 when unmarried men were accounted for, which is nevertheless a significant conclusion clearly expressing that men with children or fertile men had lower chances of future TC development. Furthermore, the study showed that increasing number of children had no impact on a potential further decrease in risks. Walsh *et al.* [2] conducted a patient study in California, US, using data collected from 22,562 patients. This pool included infertility cases due to male factors, female factors, as well as combined factors. The analysis identified men with male factor infertility using semen analysis. Using SEER the study compared specific findings of interest with data from an average population, matching categories of age and geographical location in order to close the margin for error. The authors found that infertile patients with male factor infertility were at a 2.8 higher risk for eventual TC development compared to the average population, validating conclusions from previous studies. Data from studies examining the relationship between testicular cancer and male infertility are summarized in Table 1.

**Table 1:** Epidemiologic studies focused on the association between male infertility and testicular cancer.

Author(s)	Country and Year	Design	Subjects	Finding(s)	Conclusions
Pryor *et al.* [129]	UK 1983	Case study	2043 males from infertile couples who underwent testicular biopsy from 1955 to 1982.	Carcinoma *in situ* (CIS) was diagnosed in 8 men (0.39%). 6 patients with CIS cells developed testicular tumors, one remained tumor-free and one was lost to follow-up.	The findings are applicable to the selection of patients for biopsy and appropriate treatment of CIS when diagnosed.
Strader *et al.* [136]	Western Washington State, USA 1988	Population based case-control study	Patients diagnosed with TC between 1977 and 1983 (*n* = 333) and 675 healthy controls.	Men with a history of cryptorchidism were 5.9 times more likely to develop TC than men without such history. Men with unilateral cryptorchidism were at a greater risk of tumor development on the side of the nondescent testicle (relative risk of 8.0) than on the opposite side (relative risk of 1.6). The risk tended to be smaller among cryptorchidic men who had undergone orchiopexy before adolescence.	The study supports the hypothesis that one or more local factors may account for the increased risk of germ cell testicular tumors in cryptorchidic men.
Møller and Skakkebæk [137]	Denmark 1999	Population based case-control study	514 patients diagnosed with TC identified in the Danish Cancer Registry and 720 controls randomly selected from the Danish population.	A reduced risk of TC associated with paternity (odds ratio of 0.63). Patients who before TC had a lower number of children than expected, faced a relative risk of 1.98. No corresponding protective effect associated with a higher number of children than expected was found. Similar associations were recorded for seminoma and non-seminoma cases.	Data supporting the hypothesis that compromised male fertility and TC share important etiologies.
Jacobsen *et al.* [132]	Denmark 2000	Cohort study	3530 Danish men, born between 1945–1980 and diagnosed with TC in the period of 1960–1993. Control: the total population of Danish men born between 1945–1980 (*n* = 1,488,957) and their biological children (*n* = 1,250,989).	Men, who developed TC, had a reduced fertility prior to the diagnosis (odds ratio of 0.93). A significantly lower proportion of boys was born to the patients when compared with the general population. The reduction in fertility was more pronounced in men with non-seminoma. The reduction in offspring sex ratio was independent of the TC type.	The study confirms earlier results from less conclusive studies, and indicates that TC, subfertility and a female-biased sex ratio among newborns are interrelated by biological mechanisms.
Jacobsen *et al.* [134]	Denmark 2000	Cohort study	32,442 men who had a semen analysis done during 1963–1995.	Patients with fertility issues were more likely to develop TC than other men (89 cases, incidence ratio of 1.6). The risk was relatively constant with increasing time between semen analysis and cancer diagnosis. Low semen concentration (incidence ratio of 2.3), poor spermatozoa motility (2.5), and high incidence of morphologically abnormal spermatozoa (3.0) were all associated with an increased risk of TC.	The results emphasize on the existence of common etiologies for low semen quality and TC. Low semen quality may be associated with increased incidence of germ cell tumors.
Pasqualotto *et al.* [138]	Cleveland, USA 2003	Case study	Seven patients presenting with infertility, followed by eventual TC diagnosis over a 15-year period.	Two men had elevated serum follicle stimulating hormone and luteinizing hormone levels, 1 an abnormally low serum testosterone level prior to the TC diagnosis. Tumor markers were normal in all patients. The tumor was found on the right side in 4 patients and on the left in 3. 5 cases presented with a seminoma, 1 with Leydig cell tumor and 1 carcinoma *in situ*. Follow-up on fertility status was available in 6 cases, only one patient established a pregnancy.	Most of the men who have TC and male infertility will most likely present with a seminona. Men diagnosed with infertility should be thoroughly investigated to rule out diseases associated with their infertility.
Richiardi *et al.* [139]	Sweden 2004	Population based case-control study	4592 patients with TC and 12,254 control subjects.	Before diagnosis, TC patients had lower number of children (odds ratio of 0.71), with a lower frequency of dizygotic twinning (odds ratio of 0.49). Increased occurrence of twinning after diagnosis, probably due to treatment for iatrogenic infertility.	The report provides evidence of an association between subfertility and the subsequent risk for TC.
Doria-Rose *et al.* [135]	Western Washington State, USA 2005	Case-control study	329 TC patients diagnosed from 1977 to 1983, and 672 cancer-free controls.	Decreased TC risk in men who had previously fathered a child (odds ratio of 0.76). Previous diagnosis of infertility was associated with an increased risk of TC (odds ratio of 2.40).	The results are consistent with an increased risk of TC among men with reduced fertility, going beyond the effects of cryptorchidism.
Walsh *et al.* [2]	State of California, USA 2009	Cohort study	A total of 51,461 couples evaluated for infertility from 1967 to 1998 linked with 22,562 TC patients.	34 post-infertility-diagnosis cases of TC were identified. Men seeking infertility treatment had an increased risk of subsequently developing TC (incidence ratio of 1.3), along with a markedly higher risk among those with known male factor infertility (odds ratio of 2.8).	Men with male factor infertility have an increased risk of subsequently developing TC, suggesting common etiologic factors for infertility and TC.

Infertility has been studied as a risk factor for PC development in the past. The most acknowledged report interrelating male infertility with prostate cancer incidence is a study by Walsh *et al.* [140] focused on men treated in 15 clinics located in the state of California, US, and collecting data from 22,562 cases, 4549 of which had male factor infertility. The results showed that a larger proportion of men with male factor infertility developed prostate cancer compared with those without (1.2% *vs.* 0.4%) with an average time period from infertility evaluation to cancer diagnosis of 11 years. A total of 168 cases ultimately developed PC. This number was lower than the population standard, which was expected to reach 185 cases, suggesting that all patients in the cohort were at a lower risk for PC diagnosis. No significantly elevated chances for low grade PC occurrence was associated with patients having male factor infertility, while a 2-fold increase in the PC risks was recorded for high grade PC. Furthermore, men without male factor infertility were generally at a lower risk for PC when compared to the population, showing a 0.7 decrease in low grade PC, and a 0.8 decrease in high grade PC. When studying the duration of infertility treatment, men with male factor infertility were found to have 1.8 times the hazard of any PC development compared with those without male factor infertility, furthermore, they were 2.6 times more likely to develop high grade PC. An interesting finding was that for every year of male infertility treatment the patients were 1.2 times more likely to develop PC, meaning that after 5 years of treatment the patients were facing a 2-fold increase in the probability of PC development. Furthermore the study was able to conclude that each additional year to the age was accompanied by a 10% increased risk for PC development. Walsh *et al*., admit that the results could be slightly biased, as men with male factor infertility were more likely to be screened for prostate cancer. They justify this conclusion by pointing out that the data did not cause substantial changes to the final result due to lack of significant differences in the low grade PC among men with male factor infertility [140].

On the other hand, a variety of studies pointed out that reproductive dysfunction could in fact be associated with a lower risk of PC development in infertile men. Diverse experiments connecting the effects of androgens in cell growth and proliferation [141,142] showed that decreased levels of androgens in animal models were associated with a decreased PC risk [143], failing to prove that elevated androgen production could be associated with an increased risk for a prostate malignancy [144]. This supports the idea of an androgen saturation model, according to which there is a point of a maximum threshold for androgens above which, the chances of PC development significantly decrease [145]. One of the most influential and reliable studies elaborating on this hypothesis was conducted by Ruhayel *et al.* [46] in Sweden, based on a large cohort of subjects according to the data obtained from the Swedish National Cancer Registry. The experimental design included 445 prostate cancer patients together with 446 controls and the hypothesis was based on the assumption that men with chronic testicular dysfunction would produce less androgens, thus face a decreased risk for a prostate malignancy. All childless men due to their free will or female factor infertility were excluded. Consistent with this hypothesis was the revelation of only two cases of non-fatal prostate cancer in an epidemiological study including 3518 men with the Klinefelter syndrome [8]. This result was lower than average chances for a PC diagnosis and can be contributed to the hypogonadism that is commonly observed in these patients [47]. Since a large number of infertile men had hypogonadism [96,146] thus produced less androgens, their chances for PC development were significantly lower. The general conclusion of the study was that infertile men had half the risk for developing PC compared to men with proven fertility. Previous test trials have also supported this result, as Thompson *et al.* [147] showed that patients treated with inhibitors of the testosterone conversion had a 25% lower incidence of a prostate tumor. Andriole *et al.* [148] used a similar inhibitor and found a 23% lower probability of PC development. A study by Jørgensen *et al.* [149] in Denmark used information gathered from the national population-based register, looking at the associations between infertility and PC. The team analyzed a pool of 3400 prostate cancer patients, concluding that childless men had a 16% (0.84) lower risk of PC development compared with men who had at least 1 child. A Swedish study by Giwercman *et al.* [150] looked into the link between male testicular function and PC development. In an attempt to prove this hypothesis they conducted a nationwide population-based case-control study, through which they were able to identify all 48,910 PC patients born from 1916 onwards. The study hypothesized that childless men were less likely to develop PC when compared to men who have fathered children. The results showed a reduced risk of 0.83 in childless men compared to men with ≥2 children. The authors also believe that poor semen parameters are indicative of testicular dysfunction, which could be a protective measure against prostate cancer in the reproductive age. At the same time the authors acknowledge that their analysis of childless subjects alone may not represent an accurate measure for the effects of male factor infertility on eventual PC occurrence. Instead, they note that by reducing the category of childless men to patients with male factor infertility the results would indicate a further reduction of the risk for PC development. Furthermore, the study showed that the degree of fertility or infertility was associated with PC risks, as men with 0 children were facing a 0.83 odds ratio, men who fathered 1 child closely followed with a value of 0.93, and men with >2 children were facing a 1.0 ratio, defined as the reference value. Studies examining the relationship between prostate cancer and male infertility are summarized in Table 2.

**Table 2:** Epidemiologic studies of the association between male infertility and prostate cancer.

Author(s)	Country and Year	Design	Subjects	Finding(s)	Conclusions
Giwercman *et al.* [150]	Sweden 2005	Population-based case-control	48,850 cases of PC between 1958–1998. For each case, one control was matched by year of birth.	Men being childless or having fathered one child only were associated with reduced risks for PC compared to cases having fathered 2 or more children (odds ratio of 0.83 and 0.93; respectively). There was no further change in risk associated with fathering of more than 2 children. The risk for PC was reduced among childless men.	A dysfunctional reproductive system supporting the prostatic growth to a lesser extent could be a feasible underlying cause of this association.
Negri *et al.* [151]	Italy 2006	Case-control study	1294 patients diagnosed with PC between 1991 and 2002, and 1451 controls as cases for a wide spectrum of acute and non-neoplastic conditions.	Compared to men with 2 or more children, the odds ratio for childless men was 0.95 when adjusting only for age and geographic locality, and 1.10 after further adjustment for marital status and age at marriage. The odds ratio was adjusted to 1.00 when unmarried and separated/divorced men were accounted for, 1.09 in terms of men below 65 years of age and 1.13 with respect to cases above the age of 65 years. The odds ratio was 1.17 for men with only 1 child when compared to men who reported 2 or more children.	The report concludes that the relation between the number of children and PC risk remains controversial.
Haralp *et al.* [152]	Israel 2007	Cohort study	15,268 fathers followed for 28–41 years from the birth of a live offspring.	543 men with one or more stillborn offspring experienced an increased risk of PC (incidence ratio of 1.87). With one reported stillbirth, the risk ratio was 1.68 and with two or more, the risk ratio was 3.29.	The study suggests that stillbirth and PC may have shared environmental causes. Genetic susceptibility to PC might increase the risk of a stillbirth in offspring.
Jørgensen *et al.* [149]	Denmark 2008	Cohort study	All men born in Denmark between 1935 and 1988, among whom 3400 developed PC during follow-ups between 1968 and 2003.	Childless men were at a 16% reduced risk of PC compared with fathers (incidence ratio of 0.84). The sex of the offspring did not affect PC risk (odds ratio of 0.99). Among fathers, a significant trend was observed of gradually reduced PC risk with the increasing number of children.	Men without children are at a moderately reduced risk of PC. Among men with children, there appears to be a linear decline in PC occurrence with an increasing number of children, independent of the sex of the offspring.
Ruhayel *et al.* [46]	Sweden 2010	Case-control study	445 PC cases and 446 controls. 841 men were biological fathers and 50 men were infertile.	Infertile men were at a significantly lower risk of being diagnosed with PC than fertile men (odds ratio of 0.45).	Enduring male infertility may be associated with a reduced PC risk, validating the theory that normal testicular function and steroidogenesis are important factors to the later development of PC.
Walsh *et al.* [140]	State of California, USA 2010	Population-based case-control	A total of 22,562 patients being evaluated for infertility from 1967 to 1998, and linked to the cancer registry. The incidence of PC was compared with the incidence in an age- and geography-matched sample of men from the general population.	168 cases developed PC development after infertility diagnosis. Men evaluated for infertility but not specifically with male factors were not found to have an increased risk of cancer compared with the general population (incidence ratio of 0.9). The highest risk was found in cases with male factor infertility who developed high–grade PC (incidence ratio of 2.0). According to a multivariate analysis, men with male factor infertility were found to be 2.6 times more likely to be diagnosed with high–grade PC.	Male infertility may be an early and identifiable risk factor for the development of clinically significant PC.
Wirén *et al.* [153]	Sweden 2013	Population-based case-control	117,328 PC cases and 562,644 controls, matched on birth year and residence.	Childless men had a decreased risk of PC when compared to fathers (odds ratio of 0.83) and the risk was lower for low-risk PC (odds ratio of 0.74) than for metastatic PC (odds ratio of 0.93). Adjustment for marital status and education narrowed the ratio in the low-risk category (0.87) whereas the odds ratio for metastatic cancer remained almost unchanged (0.92).	The report claims that associations between the fatherhood status and PC are predominantly due to socioeconomic factors influencing health care-seeking behavior.

## 5. Clinical Potential of MicroRNAs in the Diagnosis and Treatment of Male Infertility and Reproductive Cancers

Molecular biomarkers are a new and promising strategy to improve noninvasive diagnostics of male reproductive disorders, facilitating their management through effective screening, early diagnosis and more accurate prognosis. Furthermore these molecules may be more abundant in semen than in blood or urine, thus they may be more easily identified and quantified using PCR, RT-PCR or mass spectrometry. Biomarkers of male infertility, testicular or prostate cancer are now emerging, and it is indisputable that semen analysis through genomics and proteomics has the potential to complement other diagnostic tools available in urology and andrology clinics [154].

MicroRNAs (miRNAs) are non-coding single-stranded RNA molecules of about 18–22 nt that play important roles in regulating posttranscriptional gene silencing via base pair binding to the 3' untranslated region of their target messenger RNAs (mRNAs) [155,156]. Regulating the expression of more than 60% of genes responsible for protein encoding, miRNAs are involved in almost every biochemical process in the organism, hence their proper function is pivotal for a normal cellular development [157]. Changes in the expression patterns of miRNAs could therefore affect gene transcription and/or translation, leading to a compromised spermatogenesis, or to the occurrence of several types of malignancies, including testicular or prostate cancer [157,158].

Preliminary studies have reported that miRNAs such as miR-18a [159], miR-122a [160] and the miR-34 family [158,161] are emerging as key players in germ cell function and cell fate determination, acting to interpret and transduce cellular signals in order to allow the maintenance of undifferentiated stem cell populations, while on the other hand allowing cell differentiation during spermatogenesis. These fundamental roles for miRNAs in germ cell development have implications for normal and disease states such as infertility and germ cell tumors [158].

Testicular cancer has a unique miRNA expression profile, and several miRNA molecules have been implicated in its neoplastic development, e.g., miR-372 and miR-373 [162,163]. The first report suggesting interactions between male infertility and TC via miRNAs was published by Voorhoeve *et al.* [162]. According to this study small RNAs derived from novel oncogenes Mirn322 and Mirn323 could mediate the expression of mRNAs derived from these genes, hence they could play a role in developing testicular germ cell tumors [162]. Subsequently a high-throughput microRNAome analysis in human germ cell tumors revealed that the expression profiles of 156 miRNAs differed in type II and type III TC subjects suggesting the importance of miRNA in male infertility due to a testicular malignancy in some cases [163]. A novel molecular connection between male infertility and TC has been proposed via miR-383 regulation [164,165]. miR-383 expression has been shown to be downregulated in the testes of infertile men with maturation arrest. At the same time, downregulation of this small RNA results in enhanced proliferative activity of germ cells. While a direct target of miR-383 is the interferon regulatory factor-1 (IRF1), which has been identified as a tumor-suppressor gene, it seems to have a pro-mitogenic role in spermatogonia and early spermatocytes. The inhibition of IRF1 as a result of miR-383 activity leads to reduced levels of signaling molecules exhibiting antiproliferative and tumor-suppressor effects. Thus, disruptions of the miR-383 expression may lead to spermatogenic failure as well as promotion of testicular carcinoma cell proliferation [165].

To date, most of the studies related to the roles of microRNAs in prostate cancer highlight the potential of miR-141, miR-200b and miR-375 as significant disease correlates, which could potentially be used in tests at the time of PC diagnosis [166]. It is important to note that there appears to be a complex interaction between androgen signaling in PC, microRNA expression, and various key pathways in prostate tumorigenesis. In essence, certain miRNAs have been shown to be regulated by androgen-receptor (AR) mediated signaling while others are involved in modulating the function of the AR signaling pathway, providing additional evidence to an intricate endocrine interplay in the male reproductive system [167]. MiR-125b has emerged as a prominent androgen-responsive microRNA molecule whose upregulation may result in androgen-independent growth of prostate tumors, most likely through its anti-apoptotic effects [168]. A substantial interest has risen with respect to the miR-34 family—a group of putative tumor suppressors, which were originally found to be a direct target of p53. According to Cheng *et al.* [169] suppression of these molecules leads to an expansion of the prostate stem cell compartment and the development of early invasive and high-grade prostatic intraepithelial neoplasias. Consistently, a combined deficiency of p53 and miR-34 resulted in an acceleration of self-renewal, and motility of prostate stem/progenitor cells, providing a direct genetic evidence emphasizing that miR-34 deserves further examinations with respect to its roles as a key component of prostate stem cell compartment regulation, aberrations of which may lead to cancer occurrence [169]. In the meantime, miR-18a, related to the occurrence of testicular cancer, has been shown to be upregulated in clinical prostate tumor specimens and cancer cell lines as well. miR-18a knockdown decreased cell growth in PC cells, and significantly reduced prostate tumorigenesis in *in vivo* nude mice through apoptotic mechanisms, thus it may represent a therapeutically appealing option for PC treatment [170].

Given the redundancy of miRNAs, there is a strong prospect for miRNAs to be involved in driving and coordinating the expression of hallmark characteristics related to altered spermatogenesis and reproductive cancers. Therefore, understanding the role of various miRNAs at different stages of a pathological growth, along with their individual expression patterns, may provide vital information to the search for prognostic biomarkers and discover potential therapeutic targets [167]. There are essentially two strategies by which miRNA molecules could be targeted to treat diseases—either through a reduction of the expression or effects of a specific miRNA, or through the induction of a pathology-suppressing miRNAs within affected cells [171]. Nevertheless, prior to taking advantage of the miRNA potential in male infertility or cancer treatment, several issues must be overcome. One of the major obstacles is the delivery and significant expense of artificially modified nucleic acids. While frequent injections could be a possible treatment option for oncological cases, this is unlikely to be used in subfertile but otherwise healthy individuals [157]. Targeting of miRNA mimics and anti-MiRs remains another limiting factor in the viability of miRNA treatment, primarily due to the fact that miRNA molecules have a multitude of functions within various tissues. Meanwhile, the use of specific lipid or polymer-based nanoparticles, adenoviral or lentiviral vectors to deliver miRNA mimics has provided promising results in a successful delivery and long-term expression within specific cell types [171]. Summarizing, as the miRNA field advances, new methods of studying specific characteristics and behavior of microRNAs in a biological system, coupled with recent biomedical progress achieved with therapeutic miRNAs using nanotechnologies is encouraging and it is expected to initiate a real clinical development of therapeutic miRNAs soon [172].

## 6. Conclusions

In conclusion, a continually increasing male reproductive dysfunction, followed by the incidence of infertility or male reproductive cancers, has become a worldwide concern. All these health conditions may be contributed to a combination of genetic, epigenetic, environmental, and perinatal causes. The exceptional sensitivity of the spermatozoon may turn male infertility into an indicator of other pathologies, including testicular or prostate cancer. It is currently widely accepted that testicular cancer incidence increases in infertile men, as it can be attributed to innate CIS cells, which may later proliferate into TGCC cells. With respect to prostate cancer, the hypotheses successfully applied in case of testicular cancer still remain highly controversial and debatable because of existing conflicting and contradictory results, thus a definite image of how male reproductive health may predict the risk for a prostate malignancy has not yet emerged. To date, some of the common etiologies applicable to male infertility and prostate cancer remain hypothetical but provide a solid foundation for future research in molecular genetics and epigenetics, eventually allowing a deeper understanding of the mechanisms driving this relationship.
